# Association of depression with keratitis: A bidirectional 2-sample Mendelian randomization study

**DOI:** 10.1097/MD.0000000000048882

**Published:** 2026-05-29

**Authors:** Yujian Zhang, Donghua Niu, Mengjie Chen, Min Ji, Junfang Zhang, Rongwei Zhou

**Affiliations:** a Eye Institute, Affiliated Hospital of Nantong University, Medical School of Nantong University, Nantong, Jiangsu, China; b Department of Respiratory and Critical Care Medicine, Nantong Third People’s Hospital, Affiliated Nantong Hospital 3 of Nantong University, Nantong, Jiangsu, China; c Department of Respiratory and Critical Care Medicine, Shanghai Sixth People’s Hospital Affiliated to Shanghai Jiao Tong University School of Medicine, Shanghai, China.

**Keywords:** causal association, depression, keratitis, Mendelian randomization, risk factor

## Abstract

Observational studies suggest a potential link between depression and keratitis, but the causal relationship is unclear due to confounding. We used Mendelian randomization (MR) to examine the bidirectional causality between them. A bidirectional 2-sample MR analysis was performed using publicly available summary-level data from genome-wide association studies (GWAS) for depression and keratitis. The primary analytical method was the inverse-variance weighted (IVW) approach using a random-effects model. To ensure robustness of the findings, this was supplemented with 3 additional MR methods: weighted median, simple mode and weighted mode. Several sensitivity analyses were conducted, including Cochran *Q* test to assess heterogeneity, the MR-Egger intercept test to detect horizontal pleiotropy, and leave-one-out analysis to determine the influence of individual genetic variants. The IVW analysis showed a significant positive causal effect of depression on keratitis risk (odds ratio: 1.173; 95% confidence interval: 1.031–1.333; *P* = .015). Supplementary MR methods had consistent effect directions. Sensitivity analyses supported the primary result, showing no significant heterogeneity (Cochran *Q* = 19.194, *P* = .509) or horizontal pleiotropy (MR-Egger intercept = 0.011, *P* = .433). Leave-one-out analysis confirmed no single instrumental variable drove the causal estimate. Reverse MR analysis found no causal effect of keratitis on depression (*P* > .05). Our findings support a unidirectional causal relationship, with depression conferring an increased risk of keratitis but no evidence for a reverse causal effect. These results highlight the importance of psychiatric assessment in the management of patients with keratitis and imply shared underlying mechanisms that merit further investigation.

## 1. Introduction

Depression is one of the most prevalent and debilitating psychiatric disorders worldwide. It is projected to become the leading cause of disability worldwide by 2030.^[1]^ The pathogenesis of depression is multifactorial, involving genetic susceptibility, environmental triggers, microbial dysbiosis, and immune dysregulation.^[2,3]^ Current therapeutic strategies are often suboptimal, and a substantial proportion of patients fail to achieve complete remission. Moreover, depression is associated with an increased susceptibility to bacterial and viral infections; nonpsychiatric comorbidities are the primary causes of hospitalization among affected individuals.^[4,5]^ Stress-induced mood disturbances may further increase vulnerability to infections and recurrent illnesses.^[6]^ The association between depression and autoimmune diseases also suggests shared pathophysiological mechanisms that bridge peripheral and central immune responses.^[7-9]^

Keratitis, a common ocular surface disorder, can result from infections or autoimmune dysregulation. Various pathogens (including bacteria, viruses, and fungi), dry eye syndrome, as well as immune-mediated inflammation, are common causes of corneal damage and vision loss.^[10-13]^ Notably, infections and autoimmune dysregulation are not only key drivers of keratitis but also exhibit potential mechanistic overlaps with depression. Furthermore, adjunctive antidepressant therapies have demonstrated efficacy in patients with reduced tear production.^[14]^ Clinical evidence suggests that improvement of ocular symptoms in exposure keratitis is associated with mood improvement,^[15]^ supporting the possibility of a bidirectional interaction between depression and keratitis.

Although limited evidence suggests an association between depression and keratitis, a causal relationship remains unestablished. Observational studies are inherently susceptible to confounding and reverse causation, thereby limiting the ability to draw definitive conclusions regarding whether depression predisposes to keratitis or vice versa. Additionally, shared pathways, such as immune dysregulation and neuroendocrine dysfunction, may underlie both conditions, although the precise mechanisms remain elusive.^[16-18]^ Current evidence primarily comes from small-scale cross-sectional studies, which lack the robustness required for causal inference and thereby hinder the development of targeted interventions.

We employed bidirectional 2-sample Mendelian randomization (MR) to investigate the causal relationship between depression and keratitis. MR is a genetic epidemiological approach that uses genetic variants (typically single-nucleotide polymorphisms [SNPs]) as instrumental variables (IVs) to infer causality between exposures and outcomes.^[19,20]^ The MR approach was employed for 2 primary reasons. First, conducting traditional experimental studies to investigate the etiological hypotheses of interest is challenging due to ethical and practical constraints. Second, by leveraging genetic IVs derived from large-scale GWAS, the MR design minimizes residual confounding and reverse causation bias, offering more robust evidence for causal inference. Our study aims to not only shed light on the influence of depression on ocular surface health but also provide a foundation for developing integrated management strategies specifically for patients with comorbid depression and keratitis.

## 2. Materials and methods

### 2.1. Study design

**Figure 1.**
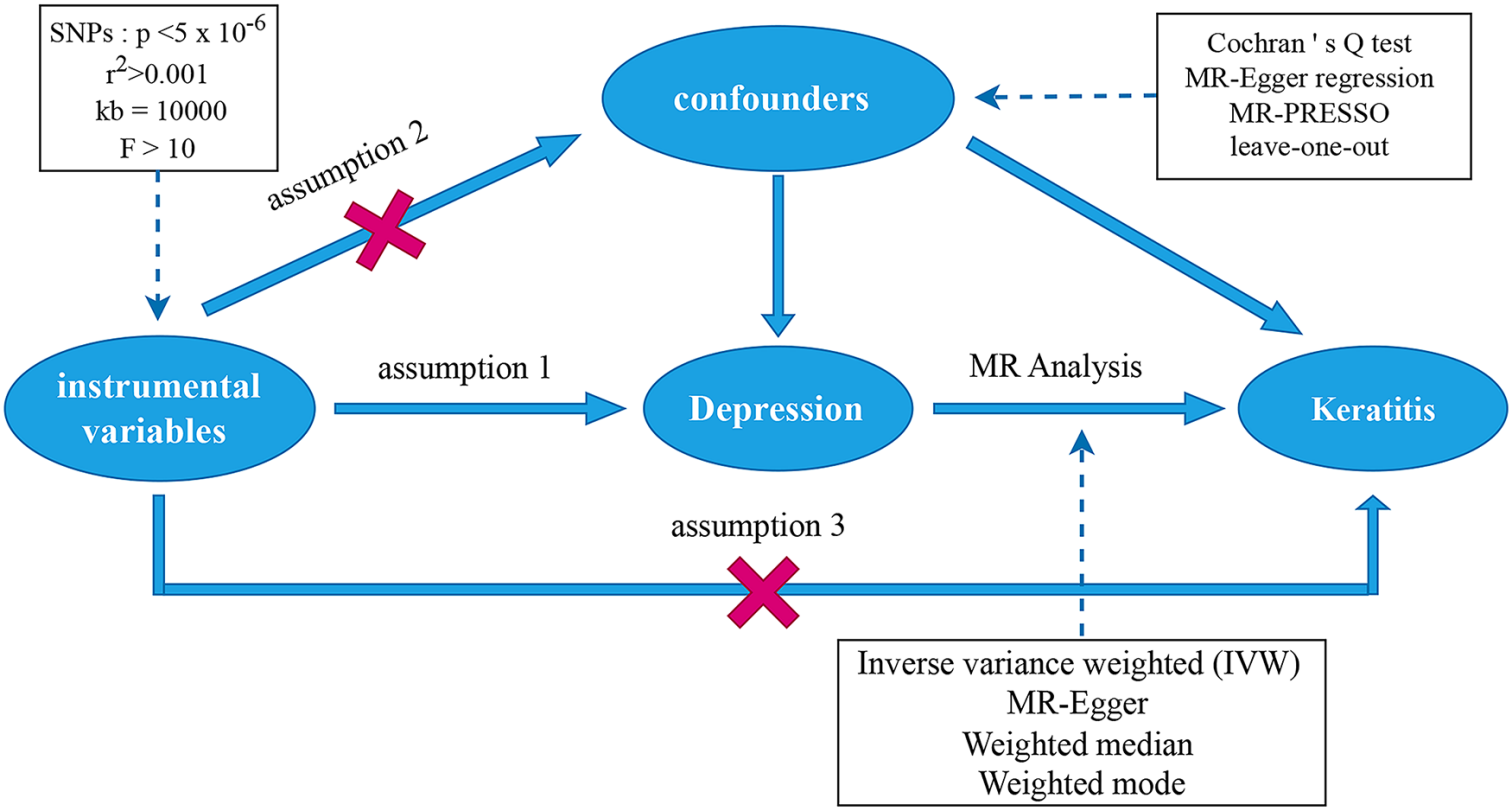
MR conceptual framework for the present study. IVW = inverse-variance weighted, MR = Mendelian randomization, MR-PRESSO = Mendelian Randomization Pleiotropy RESidual Sum and Outlier, SNP = single-nucleotide polymorphism.

A bidirectional 2-sample MR analysis was conducted to investigate the potential causal relationship between depression and keratitis using publicly available GWAS summary statistics. SNPs meeting genome-wide significance (*P* < 5 × 10^−6^) were employed as instrumental variables. All original genome-wide association studies from which data were obtained had received approval from their respective institutional review boards and documented informed consent from participants, thereby exempting the current study from additional ethical review. The overall study design is schematically presented in Figure 1. The primary analysis evaluated the causal effect of depression on keratitis risk, followed by a reverse-direction analysis assessing the effect of keratitis on depression. A comprehensive description of the MR methodology and its underlying assumptions has been published previously.^[20]^

### 2.2. Data sources

Summary statistics for the depression GWAS were sourced from the IEU Open GWAS Project (https://gwas.mrcieu.ac.uk/). This dataset included 13,559 cases and 4,35,855 controls of European descent, comprising 2,41,84,163 analyzed SNPs (GWAS ID: ebi-a-GCST90018833). These data originated from a study published in Nature Genetics that identified key genetic variants and biological mechanisms underlying disease classification.

Similarly, GWAS data for keratitis were obtained from the FinnGen Consortium (Release 10, https://www.finngen.fi/en), comprising 5561 cases and 2,09,287 controls of European descent and 1,63,80,449 analyzed SNPs (GWAS ID: finn-b-H7_KERATITIS). The FinnGen study represents a large-scale initiative that includes approximately 5,00,000 participants and employs ICD-10-based hierarchical endpoint classifications.^[21]^ All GWAS data utilized in this MR analysis were obtained from high-quality consortia, thereby ensuring genetic robustness.

### 2.3. Genetic instrument quality control

To enhance the validity of our MR analysis examining the causal relationship between depression and keratitis, stringent IV selection criteria were implemented. The IV selection process consisted of the following sequential steps: Genome-wide significance: SNPs demonstrating genome-wide significant association with the exposure (depression) at *P* < 5 × 10^−6^ were selected. Linkage disequilibrium (LD) clumping: To minimize LD bias, clumping was performed to ensure all retained SNPs exhibited pairwise *r*^2^ < 0.001 within a 10,000 kb window. Instrument strength assessment: The strength of each instrumental variable was quantified using the *F*-statistic; only SNPs with *F*-statistics > 10 were included to ensure sufficient strength against weak instrument bias. Outcome association exclusion: SNPs showing association with the outcome (keratitis) at *P* < .05 were removed to avoid potential pleiotropic effects. Additionally, SNPs not available in the outcome dataset were excluded to maintain sample overlap consistency. Data harmonization: Effect alleles were aligned between exposure and outcome datasets to ensure consistent directionality. Ambiguous SNPs (e.g., palindromic SNPs with intermediate allele frequencies) and those with allele frequency mismatches were removed.

The application of these stringent criteria yielded a final set of genetically valid IVs, thereby enhancing the robustness of the MR analysis.^[22]^ This rigorous methodological approach minimizes potential biases and strengthens the validity of causal inferences regarding the relationship between depression and keratitis.

### 2.4. MR analysis

The primary MR analysis was conducted using the IVW method to estimate the causal effect of depression on keratitis risk.^[23]^ The IVW method combines Wald ratio estimates from individual SNPs to generate an overall causal estimate, utilizing a random-effects model that provides more conservative inferences by accounting for between-SNP heterogeneity. An odds ratio (OR) >1 indicates increased risk of the outcome per unit increase in the exposure, while an OR <1 suggests a protective effect. As the IVW method assumes all genetic variants satisfy MR assumptions, we implemented complementary approaches – including MR-Egger regression, weighted median and weighted mode methods – to evaluate robustness to potential assumptionviolations.^[24-26]^ All analyses were performed using R version 4.5.1 (R Foundation for Statistical Computing) with the TwoSampleMR package.

### 2.5. Sensitivity analysis

To validate the second and third MR assumptions, comprehensive sensitivity analyses were conducted, including MR-Egger and Mendelian Randomization Pleiotropy RESidual Sum and Outlier methods.^[27,28]^ These methods complement the IVW approach by providing more robust causal estimates that are less sensitive to assumption violations. The sensitivity analyses included: Heterogeneity assessment: Cochran *Q* test was performed, with a significance threshold of *P* < .05 indicating the presence of heterogeneity. Horizontal pleiotropy assessment: MR-Egger regression was used to detect and adjust for horizontal pleiotropy; an intercept test *P*-value > .05 indicated negligible pleiotropic effects. Outlier detection and correction: The Mendelian Randomization Pleiotropy RESidual Sum and Outlier method was applied to identify and remove influential outlier SNPs, followed by distortion testing to assess pleiotropy significance. Leave-one-out analysis: Each SNP was systematically excluded iteratively to evaluate its individual influence on the overall MR estimate. Confounder screening: Potential confounding SNPs were identified and excluded using LDlink (https://ldlink.nih.gov). Following the removal of these SNPs, the IVW analysis was repeated to assess the robustness of the results.

### 2.6. The patient and public involvement statement

This MR study leveraged publicly available summary-level data from GWAS and the FinnGen database. No patients or members of the public were involved in the design, conduct, reporting, or dissemination of the research findings.

## 3. Results

### 3.1. MR analysis: effect of depression on keratitis risk

Initially, 22 independent SNPs significantly associated with depression were identified after clumping for LD at *r*^2^ < 0.001. All retained SNPs satisfied the predefined IV strength criterion (*F*-statistic > 10), confirming their suitability for MR analysis. One SNP (rs111364974) was excluded due to allele incompatibility during harmonization, yielding a final set of 21 genetically valid IVs for subsequent analyses (Table S1).

**Figure 2.**
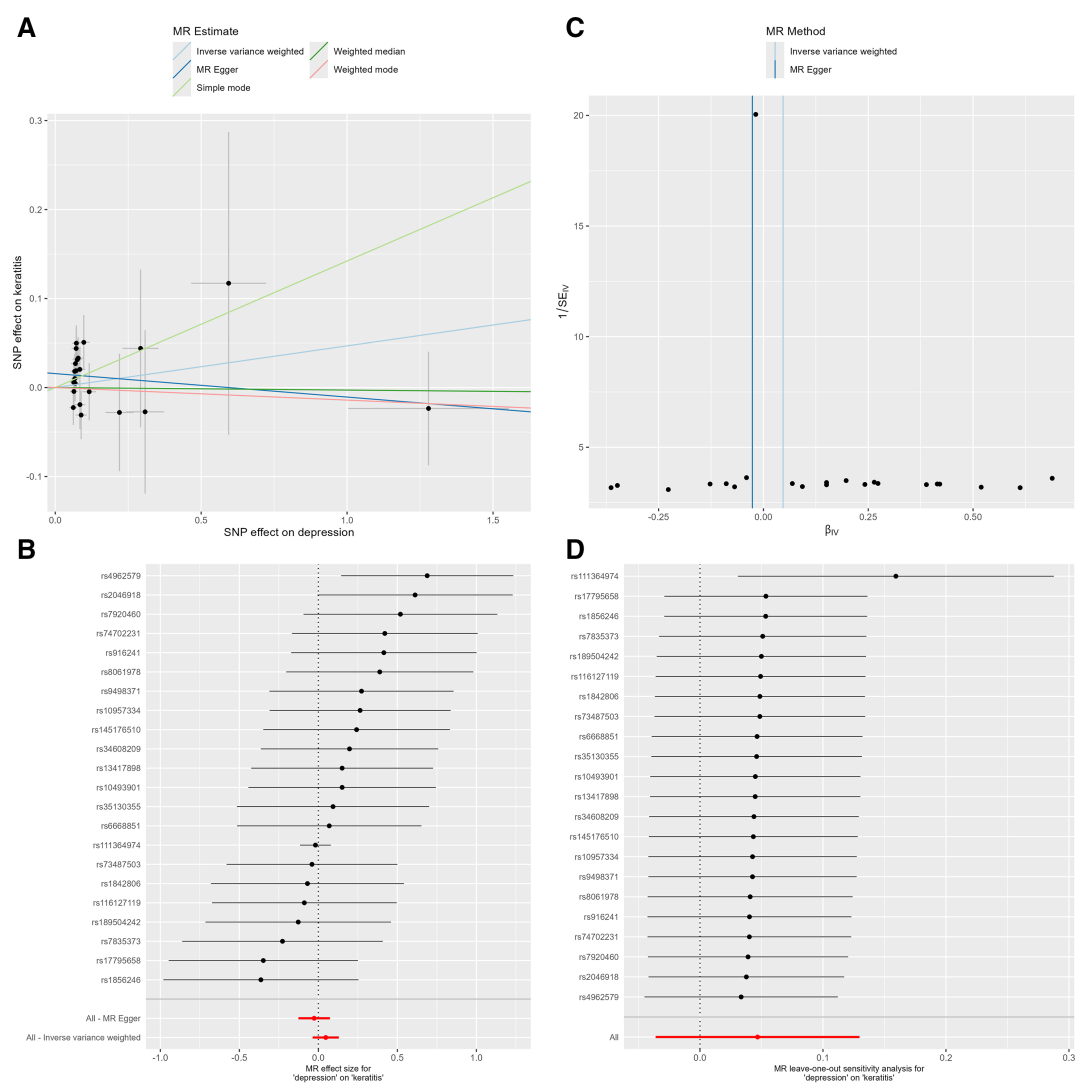
MR analyses for the association between depression and the risk of keratitis. (A) Scatter plot showing the effect of depression on keratitis. (B) Funnel plot illustrating the effect of depression on keratitis. (C) Forest plot of each SNP associated with depression on keratitis. (D) Leave-one-out plot assessing the effect of depression on keratitis. MR = Mendelian randomization, SNP = single-nucleotide polymorphism.

The IVW method demonstrated a statistically significant positive causal effect of depression on keratitis risk (OR: 1.173, 95% CI: 1.031–1.333, *P* = .015). Cochran *Q* test revealed no significant heterogeneity (*Q* = 19.194; *P* = .509), and MR-Egger regression identified no evidence of horizontal pleiotropy (intercept = 0.011; *P* = .433), collectively indicating minimal confounding bias (Table 1). Leave-one-out sensitivity analysis produced consistent results across all iterations (Fig. 2), further supporting the robustness of the causal estimate. Furthermore, LDlink analysis confirmed that no SNPs were associated with known confounding factors (Table S2). Although MR-Egger and weighted median analyses produced point estimates that diverged somewhat from the IVW estimate, the consistent directionality of effect across all genetic variants, coupled with the absence of detectable horizontal pleiotropy, supports the IVW method as providing the most reliable causal estimate in this analysis. These findings provide robust evidence for a positive causal relationship between depression and incident keratitis risk.

**Table 1:** MR results of depression on keratitis, with tests for heterogeneity and pleiotropy.

Exposure	Outcome	Method	N_snp	β	SE	OR (95% CI)	*P*
Depression	Keratitis	IVW	21	0.159	0.066	1.173 (1.031–1.333)	.015
		MR-Egger	21	0.025	0.180	1.026 (0.721–1.458)	.889
		Weighted median	21	0.151	0.095	1.162 (0.964–1.401)	.114
		Weighted mode	21	0.176	0.176	1.192 (0.844–1.683)	.329
		Heterogeneity	19.194*				.509
		Pleiotropy	0.011†				.433

*P* < .05 was considered statistically significant.

CI = confidence interval, IVW = inverse-variance weighted, MR = Mendelian randomization, N_snp = number of single-nucleotide polymorphism, OR = odds ratio, SE = standard error.

* *Q* value.

† Egger intercept.

### 3.2. Bidirectional MR analysis: effect of keratitis on depression risk

To evaluate potential reverse causation, a 2-sample MR analysis was performed using keratitis as the exposure and depression as the outcome. The sources of GWAS summary statistics and analytical methodologies were identical to those used in the forward-direction analysis. After applying the same genome-wide significance threshold (*P* < 5 × 10^−6^) and LD criterion (*r*^2^ < 0.001) used in the primary analysis, 14 SNPs were initially identified as potential IVs. Following rigorous quality control procedures, all 14 SNPs satisfied the IV assumptions and were retained for analysis (Table S3).

**Figure 3.**
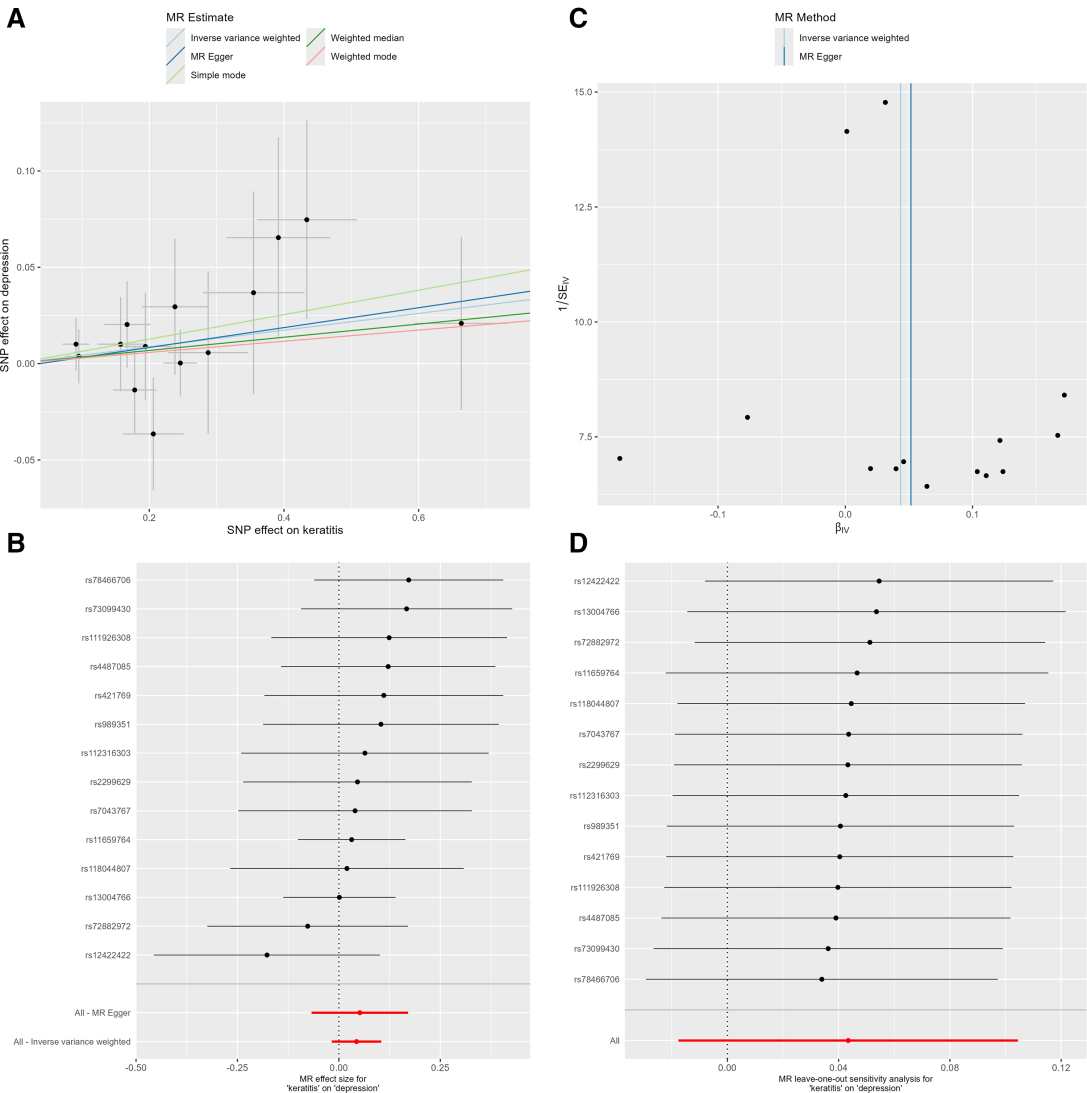
MR analyses for the association between keratitis and the risk of depression. (A) Scatter plot showing the effect of keratitis on depression. (B) Funnel plot illustrating the effect of keratitis on depression. (C) Forest plot of each SNP associated with keratitis on depression. (D) Leave-one-out plot assessing the effect of keratitis on depression. MR = Mendelian randomization, SNP = single-nucleotide polymorphism.

Notably, all MR approaches (IVW, MR-Egger, weighted median, and weighted mode) yielded null associations between keratitis and depression risk (Table 2). Cochran *Q* statistic indicated absence of substantial heterogeneity (*Q* = 6.781, *P* = .913), and MR-Egger regression revealed no significant intercept term (intercept = 0.002, *P* = .878), suggesting the absence of directional pleiotropy (Fig. 3). The consistency across methods and supporting sensitivity analyses strengthen the validity of these null findings. The reverse MR analysis revealed no significant causal effect of keratitis on depression risk, further supporting the absence of a causal relationship in this direction.

**Table 2:** MR results of keratitis on depression, with tests for heterogeneity and pleiotropy.

Exposure	Outcome	Method	N_snp	β	SE	OR (95% CI)	*P*
Keratitis	Depression	IVW	14	0.043	0.031	1.044 (0.983–1.110)	.163
		MR Egger	14	0.051	0.061	1.053 (0.935–1.186)	.412
		Weighted median	14	0.034	0.043	1.035 (0.951–1.126)	.429
		Weighted mode	14	0.029	0.055	1.029 (0.924–1.146)	.606
		Heterogeneity	6.781*				.913
		Pleiotropy	−0.002†				.878

*P* < .05 was considered statistically significant.

CI = confidence interval, IVW = inverse-variance weighted, MR = Mendelian randomization, N_snp = number of single-nucleotide polymorphism, OR = odds ratio, SE = standard error.

* *Q* value.

† Egger intercept.

## 4. Discussion

This bidirectional 2-sample MR study provides robust genetic evidence supporting a unidirectional causal relationship between depression and keratitis. Our findings indicate that genetic predisposition to depression significantly increases keratitis risk, with no evidence of reverse causality, thereby establishing depression as an independent risk factor for keratitis. To our knowledge, this represents the first MR study to comprehensively evaluate bidirectional causation between depression and keratitis using a 2-sample framework. Although observational studies have reported this association, their inherent limitations, including residual confounding and reverse causation, preclude definitive causal inference.^[15,29]^ The MR methodology employed here effectively mitigates confounding, reverse causation, and non-differential measurement error. Comprehensive sensitivity analyses further confirmed the robustness of our results, with no evidence of horizontal pleiotropy.

Although the precise mechanistic pathways linking depression and keratitis remain incompletely elucidated, shared immuno-inflammatory signaling pathways are likely involved. Notably, the pathophysiology of keratitis parallels the dual immune dysregulation characteristic of depression, which involves concurrent immunosuppression and proinflammatory activation.^[30]^ Depression is associated with upregulation of systemic proinflammatory cytokines (e.g., IL-6, TNF-α) coupled with suppression of cellular immunity, thereby increasing susceptibility to opportunistic infections.^[31,32]^ Chronic low-grade inflammation associated with depression may exacerbate corneal immune responses and impair epithelial repair mechanisms.^[33,34]^ Furthermore, hypothalamic-pituitary-adrenal axis dysregulation in depression may alter tear film composition and disrupt ocular surface homeostasis, potentially predisposing to infectious keratitis.^[35,36]^ Collectively, these findings support an integrated biological framework in which depression-induced immuno-neuroendocrine disturbances facilitate keratitis pathogenesis.

The absence of a reverse causal effect from keratitis to depression is biologically plausible. Unlike depression – which involves sustained systemic immuno-neuroendocrine dysregulation – keratitis is typically an acute, localized corneal condition that resolves rapidly with standard therapy.^[37]^ As such, it rarely induces the persistent physiological stress or central nervous system remodeling that are characteristic of chronic inflammatory diseases robustly associated with depression. This distinction aligns with observational studies that demonstrate cross-sectional associations but fail to establish temporal precedence from keratitis to depression.^[15,38,39]^ Thus, our bidirectional MR analysis reinforces that the observed causal relationship operates specifically from depression to keratitis within the integrated biological framework we have proposed.

The clinical implications of our findings encompass 3 principal domains: First, routine depression screening should be implemented for patients with keratitis, particularly those with recurrent or severe presentations, given the established causal relationship. Second, treatment strategies for patients with comorbid depression and keratitis should prioritize antidepressants with anti-inflammatory properties (e.g., minocycline), which may reduce keratitis recurrence through dual anti-inflammatory and antimicrobial mechanisms.^[40]^ Third, integratedophthalmology-psychiatry care models should be developed, as effective management of underlying depression may improve keratitis treatment outcomes and enhance patients’ quality of life.^[41]^ To address this, integrated ophthalmology-psychiatry care models should be explored. These could include psychotherapeutic approaches to manage depression, coupled with tailored counseling to improve medication adherence and self-care practices specific to keratitis. The unidirectional nature of this relationship indicates that although keratitis treatment alone is unlikely to ameliorate depression, comprehensive management of comorbid depression remains clinically essential for optimizing patient outcomes. These insights provide an evidence base to inform the development of interdisciplinary clinical guidelines.

Despite the methodological rigor of our bidirectional MR design and the consistency of sensitivity analyses, several limitations should be acknowledged. First, residual weak instrument bias may persist if the selected genetic instruments explain insufficient phenotypic variance of depression. Second, the exclusive use of GWAS data from European-ancestry populations limits the generalizability of our findings to other ethnic groups. Third, heterogeneity across keratitis subtypes (e.g., infectious vs autoimmune) could not be assessed due to the unavailability of subtype-specific GWAS summary statistics. Fourth, although MR reduces confounding, residual pleiotropic effects through alternative pathways (e.g., shared genetic risks with autoimmune disorders) cannot be entirely excluded. Finally, the potential direct effects of antidepressant medications on keratitis risk require further investigation in specifically designed studies.

Future prospective cohort studies should evaluate whether effective treatment of depression reduces the incidence of keratitis. Mechanistic studies are needed to elucidate the immune-neural pathways underlying the depression-keratitis comorbidity. Future studies should expand to multi-ancestry GWAS and employ multivariable MR or tissue-specific expression quantitative trait locus analyses to refine causal estimates and explore potential mediating mechanisms.

This MR study provides robust evidence that depression is a modifiable risk factor for keratitis, with no evidence of reverse causation observed. These findings support the integration of mental health management into ophthalmic clinical practice and provide new insights into brain-ocular axis regulatory mechanisms.

## 5. Conclusions

This MR study provides strong genetic evidence for a unidirectional causal effect of depression on keratitis using a rigorous bidirectional 2-sample approach. Our findings indicate that depression may contribute to keratitis development rather than being merely a comorbid condition. These results highlight the need to integrate psychiatric evaluation into standard keratitis management, especially in recurrent or severe cases. Therefore, addressing depression could improve ocular surface health and reduce keratitis risk, providing a rationale for integrated ophthalmology-psychiatry care models and informing relevant public health strategies.

## Acknowledgments

We thank the FinnGen team, as well as other researchers and participants, for providing publicly available GWAS data for this analysis.

## Author contributions

**Conceptualization:** Yujian Zhang, Donghua Niu, Mengjie Chen, Min Ji, Junfang Zhang, Rongwei Zhou.

**Data curation:** Min Ji.

**Formal analysis:** Donghua Niu, Mengjie Chen, Rongwei Zhou.

**Funding acquisition:** Donghua Niu, Rongwei Zhou, Yujian Zhang.

**Investigation:** Yujian Zhang, Mengjie Chen.

**Methodology:** Donghua Niu.

**Supervision:** Yujian Zhang, Donghua Niu, Mengjie Chen, Min Ji, Junfang Zhang, Rongwei Zhou.

**Writing** – **original draft:** Yujian Zhang, Donghua Niu, Junfang Zhang, Rongwei Zhou.

**Writing** – **review & editing:** Yujian Zhang, Donghua Niu, Junfang Zhang, Rongwei Zhou.






