# Supplementary material for: Association of depression with keratitis: A bidirectional 2-sample Mendelian randomization study
Source: Medicine (Baltimore). 2026 May 29;105(22):e48882. doi: 10.1097/MD.0000000000048882 (PMC13225539; doi:10.1097/MD.0000000000048882)
Supplement: Supplementary file 1 [file medi-105-e48882-s001.docx]

| **SNP** | **Effect allele** | **Other allele** | **β** | **SE** | **P** | **F** |
| --- | --- | --- | --- | --- | --- | --- |
| rs10493901 | G | T | -0.2924 | 0.0617 | 2.18E-06 | 22.4586 |
| rs10957334 | A | G | 0.0715 | 0.0144 | 7.30E-07 | 24.6539 |
| rs116127119 | A | G | 0.3078 | 0.0653 | 2.44E-06 | 22.2183 |
| rs13417898 | C | T | -0.0671 | 0.0139 | 1.39E-06 | 23.3031 |
| rs145176510 | A | G | 0.0843 | 0.0183 | 3.90E-06 | 21.2203 |
| rs17795658 | C | T | 0.0887 | 0.0191 | 3.52E-06 | 21.5665 |
| rs1842806 | T | C | -0.0639 | 0.0139 | 4.63E-06 | 21.1335 |
| rs1856246 | G | T | 0.0619 | 0.0135 | 4.73E-06 | 21.0239 |
| rs189504242 | C | A | 0.22 | 0.0478 | 4.09E-06 | 21.1831 |
| rs2046918 | A | G | 0.0717 | 0.0155 | 3.88E-06 | 21.3981 |
| rs34608209 | T | G | 0.5941 | 0.1282 | 3.61E-06 | 21.4754 |
| rs35130355 | T | C | -0.0635 | 0.0137 | 3.49E-06 | 21.4835 |
| rs4962579 | T | C | 0.0723 | 0.014 | 2.47E-07 | 26.6698 |
| rs6668851 | A | C | -0.0682 | 0.014 | 1.03E-06 | 23.7308 |
| rs73487503 | T | C | 0.1164 | 0.0233 | 5.72E-07 | 24.9571 |
| rs74702231 | T | A | 0.0787 | 0.0169 | 3.07E-06 | 21.6858 |
| rs7835373 | A | G | 0.0846 | 0.0184 | 4.21E-06 | 21.1401 |
| rs7920460 | T | C | 0.0978 | 0.0202 | 1.29E-06 | 23.4409 |
| rs8061978 | T | C | 0.0692 | 0.0151 | 4.34E-06 | 21.0018 |
| rs916241 | C | T | -0.0755 | 0.0162 | 3.15E-06 | 21.7202 |
| rs9498371 | C | T | 0.0666 | 0.0138 | 1.43E-06 | 23.2911 |

Table S1. IVs used in the MR analysis of the causal effects of depression on keratitis.

IVs: instrumental variables; MR: Mendelian Randomization; SNP: single nucleotide polymorphism; SE: standard error.
