# Supplementary material for: Association of depression with keratitis: A bidirectional 2-sample Mendelian randomization study
Source: Medicine (Baltimore). 2026 May 29;105(22):e48882. doi: 10.1097/MD.0000000000048882 (PMC13225539; doi:10.1097/MD.0000000000048882)
Supplement: Supplementary file 2 [file medi-105-e48882-s002.docx]

Table S2. Exclusion of confounding SNPs using LDlink for depression on keratitis.

| **SNP ID** | **contact lens use** | **autoimmune traits** | **income** | **ever had refractive laser eye surgery** |
| --- | --- | --- | --- | --- |
| rs10493901 | NA | NA | NA | NA |
| rs10957334 | NA | NA | NA | NA |
| rs116127119 | NA | NA | NA | NA |
| rs13417898 | NA | NA | NA | NA |
| rs145176510 | NA | NA | NA | NA |
| rs17795658 | NA | NA | NA | NA |
| rs1842806 | NA | NA | NA | NA |
| rs1856246 | NA | NA | NA | NA |
| rs189504242 | NA | NA | NA | NA |
| rs2046918 | NA | NA | NA | NA |
| rs34608209 | NA | NA | NA | NA |
| rs35130355 | NA | NA | NA | NA |
| rs4962579 | NA | NA | NA | NA |
| rs6668851 | NA | NA | NA | NA |
| rs73487503 | NA | NA | NA | NA |
| rs74702231 | NA | NA | NA | NA |
| rs7835373 | NA | NA | NA | NA |
| rs7920460 | NA | NA | NA | NA |
| rs8061978 | NA | NA | NA | NA |
| rs916241 | NA | NA | NA | NA |
| rs9498371 | NA | NA | NA | NA |

(R2 ＞0.1, P ＜0.1, base pair window: ±500000).
