# Supplementary material for: Association of depression with keratitis: A bidirectional 2-sample Mendelian randomization study
Source: Medicine (Baltimore). 2026 May 29;105(22):e48882. doi: 10.1097/MD.0000000000048882 (PMC13225539; doi:10.1097/MD.0000000000048882)
Supplement: Supplementary file 3 [file medi-105-e48882-s003.docx]

| **SNP** | **Effect allele** | **Other allele** | **β** | **SE** | **P** | **F** |
| --- | --- | --- | --- | --- | --- | --- |
| rs111926308 | T | A | 0.2381 | 0.0494 | 1.46E-06 | 23.2308 |
| rs112316303 | G | T | -0.1574 | 0.0342 | 4.23E-06 | 21.1815 |
| rs11659764 | A | T | 0.6634 | 0.0691 | 7.85E-22 | 92.1711 |
| rs118044807 | G | T | 0.2874 | 0.0593 | 1.24E-06 | 23.4891 |
| rs12422422 | C | T | -0.206 | 0.045 | 4.66E-06 | 20.9561 |
| rs13004766 | C | G | -0.2461 | 0.0249 | 4.10E-23 | 97.6842 |
| rs2299629 | A | G | 0.1942 | 0.0402 | 1.34E-06 | 23.3371 |
| rs421769 | T | G | -0.0912 | 0.0198 | 4.02E-06 | 21.2158 |
| rs4487085 | T | C | 0.167 | 0.0345 | 1.29E-06 | 23.4312 |
| rs7043767 | T | C | -0.0953 | 0.0204 | 2.94E-06 | 21.8236 |
| rs72882972 | T | A | 0.1783 | 0.0322 | 3.03E-08 | 30.6613 |
| rs73099430 | A | C | 0.3917 | 0.0772 | 3.87E-07 | 25.7438 |
| rs78466706 | T | C | 0.4339 | 0.0741 | 4.80E-09 | 34.2881 |
| rs989351 | T | C | 0.3548 | 0.0753 | 2.41E-06 | 22.2012 |

Table S3. IVs used in the MR analysis of the causal effects of keratitis on depression.

IVs: instrumental variables; MR: Mendelian Randomization; SNP: single nucleotide polymorphism; SE: standard error.
